# A Comprehensive Review of the Mechanism, Efficacy, Safety, and Tolerability of Ubrogepant in the Treatment of Migraine

**DOI:** 10.7759/cureus.48160

**Published:** 2023-11-02

**Authors:** Ibrahim M Dighriri, Shahad Nazel, Aeshah M Alharthi, Nasreen A Altowairqi, Aqeel M Albariqi, Mona A Tohari, Atheer A Alameer, Amjad K Alsaran, Fares R ALshammari, Naif F AlMutairi, Fahad M Alsubaie, Turki A Alharbi

**Affiliations:** 1 Department of Pharmacy, King Abdulaziz Specialist Hospital, Taif, SAU; 2 Faculty of Pharmacy, Princess Nourah Bint Abdulrahman University, Riyadh, SAU; 3 Department of Pharmacy, Community Pharmacy, Taif, SAU; 4 Faculty of Pharmacy, Taif University, Taif, SAU; 5 Department of Pharmacy, Bariq Primary Health Care Center, Bariq, SAU; 6 Department of Pharmacy, Alhada Armed Forces Hospital, Taif, SAU; 7 Faculty of Pharmacy, Jazan University, Jazan, SAU; 8 Department of Pharmacy, Specialized Medical Center Hospital, Riyadh, SAU; 9 Department of Pharmacy, Community Pharmacy, Al Jouf, SAU; 10 Department of Pharmacy, Maternity and Children Hospital, Buraydah, SAU; 11 Department of Pharmacy, Al Rass General Hospital, Al Rass, SAU

**Keywords:** safety, efficacy, tolerability, migraine, ubrogepant

## Abstract

Ubrogepant is an innovative medication designed for the acute treatment of migraine, a debilitating neurological condition that profoundly impairs quality of life, productivity, and social interactions. This comprehensive review assesses the efficacy, safety, tolerability, and mechanism of action of ubrogepant through a rigorous methodology, including an in-depth literature review from reputable databases like PubMed, Web of Science, Embase, Scopus, and Cochrane. Classified as a calcitonin gene-related peptide (CGRP) receptor antagonist, ubrogepant has emerged as a potential revolutionary medication for migraine treatment. CGRP is a peptide integral to migraine pathophysiology, and its blockade has demonstrated great therapeutic potential. Unlike triptans, known for their cardiovascular risks, ubrogepant lacks vasoconstrictive properties, making it a safer alternative for a broader patient population. Ubrogepant offers significant potential for pain relief, symptom reduction, and restoration of normal function during a migraine attack, and it outperforms placebo in terms of efficacy. It also presents favorable safety, with generally mild adverse drug events (ADEs), such as nausea, dizziness, and somnolence, similar to placebo effects. Consistent results from clinical trials confirm its tolerability, with minor ADEs and no safety alerts for the tested doses, indicating that ubrogepant is a safe and well-tolerated option for migraine treatment. As an effective oral medication, ubrogepant could be an alternative to traditional acute migraine treatments. Its benefits include a unique mechanism of action, rapid onset, and favorable safety profile. However, specific contraindications, such as hypersensitivity, severe hepatic impairment, concurrent use of CYP3A4 inhibitors, pregnancy or breastfeeding, and uncontrolled hypertension, require caution or avoidance of ubrogepant. Despite these limitations, ubrogepant signals a promising new direction in migraine therapeutics.

## Introduction and background

Migraines are a common neurological condition characterized by recurrent, moderate-to-severe headache pain often accompanied by nausea, vomiting, and heightened sensitivity to light and sound [1-3]. Globally, migraines are estimated to affect approximately 12-15% of the population, making them one of the most prevalent neurological disorders [3-6]. The World Health Organization (WHO) has classified severe migraines among the top 10 most disabling illnesses, underscoring the substantial morbidity associated with this condition [6,7].

The impact of migraines extends beyond the physical symptoms. People with migraines often report decreased quality of life, missed workdays, and impaired social and family life [2-4]. The economic burden of migraines is significant, with direct medical and indirect costs related to lost productivity [3,5,6]. The exact cause of migraines is not entirely understood, but it is believed to involve the activation and sensitization of central trigeminovascular neurons [2,8,9]. Some factors triggering migraines include hormonal changes, stress, and certain foods and beverages factors [1,2].

Current migraine treatments can be categorized into two main groups: abortive and preventive [10-13]. Abortive treatments, like triptans and nonsteroidal anti-inflammatory drugs (NSAIDs), aim to stop the progression of a migraine once it has started [10,12,14,15]. On the other hand, preventive treatments, which include beta-blockers, antiepileptic drugs, and Botox injections, are taken regularly to reduce migraine severity [10,13,16,17].

Ubrogepant is a novel oral medication specifically designed for the acute treatment of migraines [18,19]. It belongs to a class of drugs known as calcitonin gene-related peptide (CGRP) receptor antagonists [18,20,21]. CGRP is a peptide that has been identified as a critical player in the pathophysiology of migraines, and blocking its activity has emerged as a promising strategy for migraine [19,20,22].

The development of ubrogepant has been hailed as a significant step forward in migraine therapeutics [23]. Unlike triptans, which are contraindicated in people with certain cardiovascular conditions, ubrogepant does not have vasoconstrictive properties, making it a safer option for a broader range of patients [23,24]. Furthermore, early clinical trials have shown that ubrogepant effectively relieves migraine symptoms without the typical side effects associated with other migraine medications [22,23].

This comprehensive review aims to examine ubrogepant in treating migraines, focusing on its mechanism of action, efficacy, safety, and tolerability. As migraines burden a significant portion of the global population, understanding the potential of new treatments like ubrogepant is crucial for clinicians and patients.

## Review

Methods

Study Design

This comprehensive review delves deeply into aspects of ubrogepant's action mechanism, effectiveness, safety parameters, and acceptability in managing migraines.

Literary Exploration

Our research commenced with an intensive exploration of the literature. We scoured databases, including PubMed, Web of Science, Embase, Scopus, and Cochrane, initiating wide-ranging searches using terms such as "Ubrogepant," "Migraine," "Action Mechanism," "Effectiveness," "Safety," "Tolerability," and "Clinical Evaluations." This robust methodology allowed us to accumulate a wide range of studies, from foundational to contemporary, yielding a comprehensive perspective on the subject.

Criteria for Inclusion and Exclusion of Studies

For a study to be included in this review, it had to meet several specific criteria. Firstly, it needed to center on the mechanism, efficacy, safety, or tolerability of ubrogepant in migraine treatment. The study type should be one of the following: a randomized controlled trial, cohort study, case-control study, retrospective study, review, systematic review, or meta-analysis; and it must have been published in English within the last 10 years. On the other hand, we excluded studies that were editorials, opinion pieces, or letters to the editor. Studies with ambiguous methodologies or significant methodological shortcomings were also left out.

Comparative Analysis and Synthesis of Findings

With the data in hand, a comparative analysis was executed. This permitted us to identify consistencies, variations, and potential gaps in findings across multiple studies. This analysis was crucial in comprehending the comprehensive scope of ubrogepant's application and its impacts. The gathered findings were synthesized to offer a complete overview.

Process of Searching

**Figure 1:**
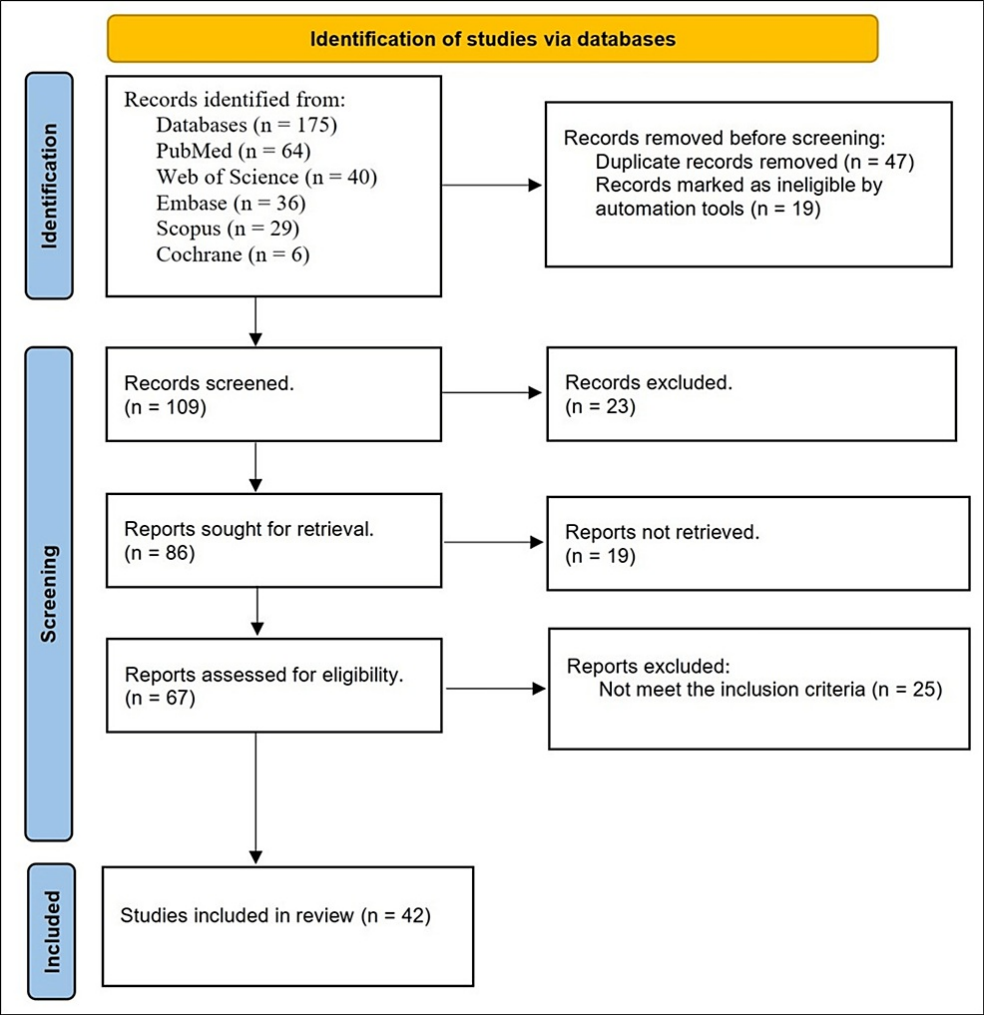
Process of identifying, screening, and including studies for this review.

Figure 1 details the process of identifying, screening, and including studies for this review. A total of 175 records were initially identified from five databases: PubMed (n = 64), Web of Science (n = 40), Embase (n = 36), Scopus (n = 29), and Cochrane (n = 6). After removing 47 duplicate records, automation tools excluded an additional 19 records as ineligible, leaving 109 records to be screened. Of these, 23 records were excluded upon screening, resulting in 86 potentially relevant reports to be retrieved. However, 19 reports could not be retrieved, so 67 reports remained for full-text eligibility assessment. Of those, 25 reports were excluded for not meeting the inclusion criteria. Ultimately, 42 studies were included in this review.

Pharmacological profile and mechanism of action of ubrogepant

Pharmacological Profile of Ubrogepant

Ubrogepant is prescribed for treating migraines in adults, both with and without aura [25,26]. As a CGRP receptor antagonist, it counters the role of the CGRP receptor in migraine development [26,27]. CGRP is integral to transmitting pain signals and dilating blood vessels. Ubrogepant's inhibition of this receptor helps alleviate migraine symptoms [26,27].

The standard dosages are 50 mg or 100 mg, taken orally. Its pharmacokinetic attributes, guided by first-order kinetics, show that the CYP3A4 enzyme primarily metabolizes it [28-30]. A phase 1 study highlighted increased systemic exposure to ubrogepant in individuals with hepatic impairment versus those with standard liver functionality. Although no dosage adjustment is needed for mild to moderate liver impairment, those with severe impairment are advised to take 50 mg [30,31].

Ubrogepant offers pain relief within two hours of dosage during acute migraine episodes [30,32]. After administration, its peak plasma concentration is reached within an hour, sustaining therapeutic levels for about 12 hours [27,29]. This positions it as an alternative for those unresponsive to other analgesics or triptans [30,33]. Its clinical efficacy is comparable to triptans and other CGRP-targeted drugs but with enhanced tolerability over earlier triptans [33-35]. While its potential as a migraine treatment is evident, its safety needs further exploration, especially in specific groups like cardiovascular patients or expectant mothers [36,37].

Mechanism of Ubrogepant in Migraine Alleviation

**Figure 2:**
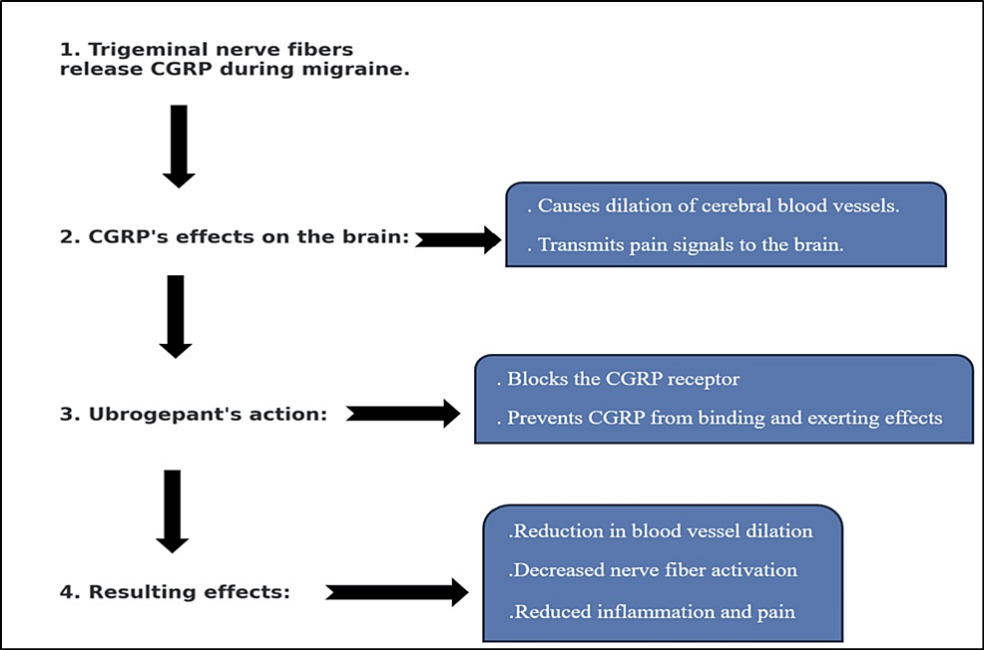
The mechanism of ubrogepant in migraine alleviation. CGRP: calcitonin gene-related peptide. References: [27,38,39].

Ubrogepant addresses migraines by intervening in the CGRP pathway. During migraine, trigeminal nerve fibers release CGRP, leading to cerebral blood vessel dilation and pain signal transmission to the brain [27,38,39]. It also prompts the release of pro-inflammatory agents, intensifying the migraine's inflammation and discomfort [35,38,39]. As a CGRP receptor antagonist, ubrogepant blocks this receptor, preventing CGRP from binding and exerting its effects. Consequently, there is a reduction in blood vessel dilation, nerve fiber activation, and the release of inflammatory agents, which overall diminishes migraine symptoms (Figure 2) [19,27,35].

Ubrogepant's efficacy

Clinical Trials on Ubrogepant's Efficacy

Ubrogepant has demonstrated significant efficacy in several clinical trials. The trials, which varied in their methodologies, provided evidence of ubrogepant's therapeutic potential. In the ACHIEVE II trial conducted by Lipton et al. in 2019, ubrogepant stood out for its ability to provide pain relief. The study found that 20.8% of patients achieved freedom from pain at two hours post-dose with either 25 mg or 50 mg of ubrogepant, compared to just 12.6% with placebo (p < 0.001) [40]. Moreover, ubrogepant also demonstrated a superior ability to provide pain relief within two hours, with 60.7% of patients reporting relief compared to 42.4% with placebo (p < 0.001) [40]. The absence of the most bothersome symptom (MBS) was also significantly higher in the ubrogepant group (37.6%) than in the placebo group (27.8%) at two hours (p < 0.001) [40].

A phase IIb trial conducted by Voss et al. in 2016 investigated a range of ubrogepant doses from 25 to 100 mg [41]. The study results indicated that the absence of phonophobia at two hours post-dose ranged from 56% to 66% with ubrogepant, compared to 48% with placebo (p < 0.05 for 50 mg and 100 mg) [41]. The absence of photophobia at two hours was also significantly higher with ubrogepant (48-55%) compared to placebo (37%; p < 0.05 for 50 mg and 100 mg) [41].

Finally, the ACHIEVE I trial conducted by Dodick et al. in 2019 provided further evidence of ubrogepant's efficacy. The study found that pain freedom at two hours was achieved by 21.8% of patients on 100 mg ubrogepant and 20.7% on 50 mg, compared to 14.3% with placebo (p ≤ 0.001 for both) [26]. Sustained pain relief from two to 24 hours was reported by 42.3% of patients on 100 mg and 34.1% on 50 mg, compared to 19.8% with placebo (p < 0.001 for both) [26]. Furthermore, return to normal function at two hours was significantly higher in ubrogepant groups (45% on 100 mg, 40.9% on 50 mg) than with placebo (29.5%; p<0.001 for both) (Table 1) [26]. Overall, results from these clinical trials collectively suggest that ubrogepant is an effective treatment option for migraines. This novel medication has shown a significant potential to relieve pain, reduce bothersome symptoms, and help patients return to normal functions more quickly than a placebo.

**Table 1:** Summary of clinical trials on ubrogepant's efficacy.

Study	Outcome	Ubrogepant 25 mg	Ubrogepant 50 mg	Ubrogepant 100 mg	Placebo	Significance
Lipton 2019 (ACHIEVE II) [40]	Pain freedom at 2 hours	20.8%	20.8%	-	12.6%	p < 0.001
	Pain relief at 2 hours	-	60.7%	-	42.4%	p < 0.001
	Absence of most bothersome symptom at 2 hours	-	37.6%	-	27.8%	p < 0.001
Voss 2016 (Phase IIb) [41]	Absence of phonophobia at 2 hours	-	Ranged from 56% to 66% (p < 0.05 for 50 mg and 100 mg)	-	48%	p < 0.05
	Absence of photophobia at 2 hours	-	Ranged from 48% to 55% (p < 0.05 for 50 mg and 100 mg)	-	37%	p < 0.05
Dodick 2019 (ACHIEVE I) [26]	Pain freedom at 2 hours	-	20.7%	21.8%	14.3%	p ≤ 0.001
	Sustained pain relief from 2 to 24 hours	-	34.1%	42.3%	19.8%	p < 0.001
	Return to normal function at 2 hours	-	40.9%	45%	29.5%	p < 0.001

Comparison of Ubrogepant's Efficacy With Other Treatments

Ubrogepant has been compared to other treatments in terms of its efficacy in various clinical outcomes. The comparison includes other migraine medications like rimegepant and lasmiditan [21,42]. Regarding the accomplishment of two-hour pain freedom, ubrogepant 100 mg exhibited an odds ratio (OR) of 1.97 compared to a placebo, which is on par with rimegepant 75 mg (OR = 2.0). However, it was less effective than lasmiditan 200 mg (OR = 2.88). When measuring the absence of MBS at two hours, ubrogepant 50 mg and 100 mg had similar ORs to lasmiditan 100 mg (OR = 1.61), lasmiditan 200 mg (OR = 1.66), and rimegepant 75 mg (OR = 1.61) [21,42].

Regarding achieving freedom from photophobia at two hours, a symptom commonly associated with migraines, ubrogepant 50 mg and 100 mg (OR = 1.45 and 1.82, respectively) were less effective than lasmiditan 200 mg (OR = 1.92). For gaining relief from phonophobia at two hours, ubrogepant 50 mg and 100 mg (OR = 1.45 and 1.30, respectively) were outperformed by lasmiditan 200 mg (OR = 1.72) and rimegepant 75 mg (OR = 1.61). When looking at the freedom from nausea at two hours, ubrogepant 50 mg and 100 mg (OR = 1.24 and 1.27) were less effective than rimegepant 75 mg (OR = 1.25) and lasmiditan 200 mg (OR = 1.15) [21,42].

However, in terms of sustained pain freedom at 24 hours, ubrogepant 50 mg and 100 mg (OR = 1.71 and 2.04, respectively) outshone lasmiditan 50 mg (OR = 1.36) but remained less effective than lasmiditan 200 mg (OR = 2.69). Lastly, for the measure of pain relief at two hours, ubrogepant 50 mg and 100 mg (both OR = 1.70) were less effective than lasmiditan 200 mg (OR = 2.18) and rimegepant 75 mg (OR = 1.89) (Table 2) [21,42]. Overall, ubrogepant has displayed varying degrees of efficacy compared to other migraine treatments. Its performance depends on the evaluated metric, indicating that patient outcomes may vary based on individual symptoms and response to treatment [43].

**Table 2:** Summary of comparison of ubrogepant's efficacy with other treatments. References: [21,42,43].

Outcome measure	Drug and dosage	Odds ratio (OR)
Two-hour pain freedom	Ubrogepant 100 mg	1.97
	Rimegepant 75 mg	2.0
	Lasmiditan 200 mg	2.88
Two-hour absence of most bothersome symptom	Ubrogepant 50 mg	1.61
	Ubrogepant 100 mg	1.61
	Lasmiditan 100 mg	1.61
	Lasmiditan 200 mg	1.66
	Rimegepant 75 mg	1.61
Freedom from photophobia at two hours	Ubrogepant 50 mg	1.45
	Ubrogepant 100 mg	1.82
	Lasmiditan 200 mg	1.92
Freedom from phonophobia at two hours	Ubrogepant 50 mg	1.45
	Ubrogepant 100 mg	1.30
	Lasmiditan 200 mg	1.72
	Rimegepant 75 mg	1.61
Freedom from nausea at two hours	Ubrogepant 50 mg	1.24
	Ubrogepant 100 mg	1.27
	Rimegepant 75 mg	1.25
	Lasmiditan 200 mg	1.15
Sustained pain freedom at 24 hours	Ubrogepant 50 mg	1.71
	Ubrogepant 100 mg	2.04
	Lasmiditan 50 mg	1.36
	Lasmiditan 200 mg	2.69
Pain relief at two hours	Ubrogepant 50 mg	1.70
	Ubrogepant 100 mg	1.70
	Lasmiditan 200 mg	2.18
	Rimegepant 75 mg	1.89

Ubrogepant's safety

The safety profile of ubrogepant has been extensively evaluated in several clinical trials, with consistent findings indicating a generally favorable safety profile. In the ACHIEVE II trial, 1,317 patients received either 25 mg or 50 mg of ubrogepant [40]. No fatalities were reported, and only three patients experienced serious adverse drug events (ADEs), which were not considered treatment-related [40]. Treatment-related ADEs were reported by 25.4% of those on ubrogepant, compared to 23.8% on placebo. Nausea (5.7% vs. 3.5%) and somnolence (3.4% vs. 2.5%) were the most common ADEs reported in the ubrogepant group compared to the placebo group [40].

The phase IIb trial featured 425 patients who received doses of ubrogepant ranging from 25 to 100 mg [41]. Similar to the ACHIEVE II trial, no deaths were reported. Three patients experienced severe ADEs that were not deemed treatment-related. Treatment-related ADEs displayed a dose-dependent pattern, with the highest incidence at 100 mg. The most common ADEs included nausea, dizziness, and fatigue [41].

In the ACHIEVE I trial, 1,266 patients received ubrogepant in doses of 50 mg or 100 mg [26]. As in previous trials, no deaths were reported. Two patients had severe ADEs that were not considered to be related to treatment. Treatment-related ADEs occurred in 21.8% of patients on ubrogepant versus 16% on placebo. The most common ADEs were nausea (3% vs. 1.4%) and somnolence (3.4% vs. 2%) (Table 3) [26]. These three clinical trials' safety findings consistently demonstrated ubrogepant's favorable safety profile. The ADEs reported were mostly mild, and no safety signals were detected across the tested doses from 25 to 100 mg. This suggests that ubrogepant is a safe treatment option for migraines with minimal side effects.

**Table 3:** Summary of the safety profile of ubrogepant.

Metric/study	Lipton 2019 (ACHIEVE II) [40]	Voss 2016 (Phase IIb) [41]	Dodick 2019 (ACHIEVE I) [26]
Number of patients	1,317	425	1,266
Dose of ubrogepant	25 mg or 50 mg	25-100 mg	50 mg or 100 mg
Deaths reported	No	No	No
Serious adverse events	3 (not treatment-related)	3 (not treatment-related)	2 (not treatment-related)
Treatment-related adverse events	25.4% vs. 23.8% placebo	Dose-dependent (highest at 100 mg)	21.8% vs. 16% placebo
Most common adverse events	Nausea (5.7% vs. 3.5%). Somnolence (3.4% vs. 2.5%)	Nausea, dizziness, fatigue	Nausea (3% vs. 1.4%). Somnolence (3.4% vs. 2%)

Ubrogepant's tolerability

Clinical trials have provided a wealth of information about the tolerability and acceptability of ubrogepant as a treatment for migraines (Table 4). In the ACHIEVE II trial, 25.4% of patients using ubrogepant reported treatment-related ADE compared to 23.8% on placebo, indicating a similar level of tolerability [40]. Most reported ADEs were nausea (5.7% ubrogepant vs. 3.5% placebo), somnolence (3.4% vs. 2.5%), and dizziness (3.2% vs. 2.5%). The higher rates observed with ubrogepant were relatively small, suggesting that the drug is generally well-tolerated [40].

**Table 4:** Comparison with the tolerability profiles of other migraine treatments. CGRP: calcitonin gene-related peptide. References: [23,40,41].

Medication class	Medication	Primary use	Tolerability/side effects
Triptans	Sumatriptan, rizatriptan	Acute migraine treatment	Generally well-tolerated; sensations of tightness in chest, neck, jaw, etc. Contraindicated for specific cardiovascular conditions.
Ergotamines	Ergotamine, dihydroergotamine	Counteract migraine-related dilation	It can lead to medication overuse, headaches, and nausea. Potential for vasoconstriction complications.
Beta-blockers	Propranolol, metoprolol	Preventive migraine treatment	Fatigue, depression, cold hands and feet. Contraindicated in asthma or certain cardiovascular conditions.
Anticonvulsants	Topiramate, valproic acid	Migraine prevention	Weight loss, cognitive side effects, tingling in hands and feet.
OnabotulinumtoxinA	Botox	Preventive for chronic migraines (every three months)	Generally well-tolerated; injection site reactions, rare spread of toxin effects.
CGRP monoclonal antibodies	Erenumab, fremanezumab	Preventive for episodic or chronic migraines	Generally well-tolerated injection site reactions.
	Ubrogepant	Acute treatment for episodic migraine	Generally well-tolerated. Mild side effects (nausea, somnolence, dizziness) indicate good tolerability and likely acceptability.

A phase IIb dose-ranging trial revealed a dose-dependent increase in ADE, yet the tolerability remained acceptable up to 100 mg. At the 100 mg dose, the most common ADEs were nausea (8.2%), dizziness (5.5%), and fatigue (4.1%) among ubrogepant patients [41]. Discontinuation rate was low at 2.5% across all doses, further highlighting the drug's tolerability [41]. In the ACHIEVE I trial, the rate of ADE was 21.8% with ubrogepant compared to 16% with placebo, confirming the drug's tolerability [23]. The most common ADEs were nausea (3%), somnolence (3.4%), and dizziness (2.8%) [23]. Discontinuation due to ADE was slightly higher for ubrogepant (2.1% for the 50 mg dose and 2.2% for the 100 mg dose) compared to placebo (1.1%), but these rates are still relatively low. Based on low discontinuation rates, the mild side effect profiles, and the similar rates of ADE compared to a placebo, the results indicate that patients are likely to tolerate ubrogepant well and find it acceptable as an acute treatment for episodic migraines [23]. However, it is important to note that direct patient-reported acceptability measures would provide more robust evidence regarding the acceptability of ubrogepant.

Several key factors can affect the tolerability of ubrogepant, as shown in Table 5. One such factor is the potential for ADEs, with nausea, dry mouth, and dizziness being most reported. Typically, these side effects are mild to moderate in severity, but they can influence the patient's drug tolerability [44,45]. Furthermore, drug interactions also play a significant role in tolerability. The enzyme CYP3A4 metabolizes ubrogepant, and its levels can be affected by drugs that inhibit or induce this enzyme. This interaction could potentially increase the side effects experienced by the patient, thereby impacting ubrogepant's tolerability [46]. In addition to drug interactions, the patient's underlying conditions can influence the tolerability of ubrogepant. Specifically, patients with severe hepatic or renal impairment may experience increased side effects due to reduced drug clearance. In such cases, dose adjustments may be required to ensure tolerability [45,47]. Contrarily, food intake does not significantly impact ubrogepant pharmacokinetics or associated side effects. Hence, ubrogepant can be taken without regard to meals, which may improve its tolerability for some patients [48,49]. Lastly, the dosage of ubrogepant is another crucial factor in its tolerability. Higher doses are associated with increased rates of ADE. Therefore, appropriate dosing is critical to balance efficacy and tolerability [48,49].

**Table 5:** Factors that affect ubrogepant's tolerability. References: [44-46].

Factor	Details
Adverse effects	The most commonly reported adverse effects of ubrogepant include nausea, dry mouth, and dizziness. These tend to be mild to moderate in severity.
Drug interactions	CYP3A4 metabolizes ubrogepant. Drugs that inhibit or induce CYP3A4 can affect ubrogepant levels and potentially increase side effects.
Underlying conditions	Patients with severe hepatic or renal impairment may have increased side effects with ubrogepant due to reduced clearance. Dose adjustments may be needed.
Food intake	Food does not have a clinically meaningful impact on ubrogepant pharmacokinetics or side effects. It can be taken without regard to meals.
Dosage	Higher doses of ubrogepant are associated with higher rates of adverse events. Proper dosing is essential to balance efficacy and tolerability.

Comparison of ubrogepant with traditional migraine treatments

When comparing ubrogepant to traditional migraine treatments, several critical points of differentiation emerge (Table 6). Firstly, the mechanism of action sets ubrogepant apart. Ubrogepant operates as a CGRP receptor antagonist, blocking CGRP receptors that play a role in the initiation and progression of migraines [19,50-52]. This differs from traditional oral migraine treatments, such as triptans, which function as vasoconstrictors or serotonin receptor agonists [53-56]. The speed of onset is another distinguishing feature. Ubrogepant begins to act within one to two hours, faster than many traditional oral treatments that may take one to four hours to offer relief [51,53]. This quick action makes ubrogepant suitable for acute treatment when symptoms start [50-52]. Another point of comparison lies in the route of administration. Ubrogepant is available as an oral tablet, taken right when symptoms appear [25,36]. While many traditional treatments also require ingestion at the start of migraine, this can be challenging during an attack. However, certain triptans are available as injections or nasal sprays, allowing for more rapid absorption [55,57]. The frequency of use is also a relevant factor. Ubrogepant is designed for occasional use, up to eight times monthly, when migraines occur [51,58]. This is on par with triptans and other oral treatments, which also have monthly limits due to the risk of rebound headaches. Cost-wise, ubrogepant may carry a higher initial price tag compared to generic triptans [59]. However, it provides the advantages of fast, convenient oral dosing for acute migraine treatment [58,60]. In summary, ubrogepant offers an additional acute migraine treatment option that operates quickly when taken orally and may present a safer side effect profile for some individuals than traditional oral treatments like triptans. Its unique mechanism of action further distinguishes it from traditional therapies [61-63].

**Table 6:** Comparison of ubrogepant with traditional migraine treatments. CGRP: calcitonin gene-related peptide. References: [50,61,63].

Feature	Ubrogepant	Traditional migraine treatments (e.g., triptans)
Mechanism of action	CGRP receptor antagonist	Vasoconstrictors or serotonin receptor agonists
Speed of onset	1-2 hours	1-4 hours
Route of administration	Oral tablet	Oral, injections, nasal sprays
Frequency of use	Up to 8 times/month	Varies, with monthly limits due to rebound concerns
Cost	Potentially higher initial cost	Lower for generic versions
Benefits	Fast and convenient oral dosing for acute treatment	Varies based on specific drugs and form

Ubrogepant's contraindications

Ubrogepant has specific contraindications that must be considered before a prescription, as shown in Table 7. Firstly, due to the risk of severe allergic reactions, it should not be administered to patients with known hypersensitivity to ubrogepant or any of its components [64]. Severe hepatic impairment is another critical contraindication, as the liver extensively metabolizes ubrogepant [60,61,65]. In patients with severe hepatic impairment, higher blood levels of the drug may occur, which could increase the risk of ADEs. Thus, ubrogepant is not recommended for use in such circumstances [25,60,64]. The co-administration of ubrogepant with potent CYP3A4 inhibitors, like ketoconazole, should also be avoided. Potent CYP3A4 inhibitors can significantly increase ubrogepant levels, raising the risk of ADEs [25,61,64].

**Table 7:** Essential contraindications and their underlying reasons for ubrogepant. References: [60,61,65].

Contraindication	Description and reasoning
Hypersensitivity	Risk of severe allergic reactions due to hypersensitivity to ubrogepant or its components.
Severe hepatic impairment	The liver metabolizes ubrogepant; higher blood levels may occur in these patients, increasing the risk of adverse effects.
Co-administration with potent CYP3A4 inhibitors	Potent CYP3A4 inhibitors can significantly increase ubrogepant levels, raising the risk of adverse effects.
Pregnancy and breastfeeding	Limited safety data in pregnancy and excretion in human milk. Use is not recommended.
Uncontrolled hypertension	Ubrogepant can cause transient increases in blood pressure. Use with caution or avoid in these patients.

Furthermore, there is limited data on the safety of ubrogepant during pregnancy or breastfeeding. The drug is excreted in human milk, so its use is not recommended during pregnancy or breastfeeding [61,64]. Lastly, ubrogepant should be used with caution, if at all, in patients with uncontrolled hypertension. This is because ubrogepant can cause transient increases in blood pressure [64]. In summary, the essential contraindications for ubrogepant include hypersensitivity to the drug, severe hepatic impairment, concurrent use of potent CYP3A4 inhibitors, pregnancy or breastfeeding, and uncontrolled hypertension. In these situations, the use of ubrogepant should either be avoided or approached with significant caution.

## Conclusions

This comprehensive review proves that ubrogepant is an effective and well-tolerated acute treatment option for migraines. Multiple clinical trials have demonstrated that 50 mg or 100 mg of ubrogepant can significantly improve pain freedom, relieve pain, reduce associated symptoms, and help patients return to normal function during migraine attacks, outperforming a placebo. The safety profile is favorable, with mostly mild ADEs, such as nausea, dizziness, and somnolence, comparable to placebo. Discontinuation due to side effects is low. While ubrogepant shows some variability in efficacy for specific symptoms compared to other acute treatments like triptans and lasmiditan, it provides the advantage of a novel mechanism of action as a CGRP receptor antagonist with oral administration and rapid onset of action within one to two hours. Based on clinical trial evidence, patients are likely to tolerate ubrogepant well. However, some contraindications exist, including hypersensitivity, severe hepatic impairment, concurrent use of CYP3A4 inhibitors, pregnancy/breastfeeding, and uncontrolled hypertension. This rigorous review demonstrates that ubrogepant is an effective and well-tolerated oral medication that can serve as an alternative to traditional treatments for acute migraine relief, offering advantages like its novel mechanism of action, rapid onset, and favorable safety profile.

## References

[1] Sacco S, Braschinsky M, Ducros A (2020). European Headache Federation consensus on the definition of resistant and refractory migraine: developed with the endorsement of the European Migraine & Headache Alliance (EMHA). J Headache Pain.

[2] Pescador Ruschel MA, De Jesus O (2023). Migraine Headache.

[3] Burch RC, Buse DC, Lipton RB (2019). Migraine: epidemiology, burden, and comorbidity. Neurol Clin.

[4] Safiri S, Pourfathi H, Eagan A (2022). Global, regional, and national burden of migraine in 204 countries and territories, 1990 to 2019. Pain.

[5] Steiner TJ, Stovner LJ (2023). Global epidemiology of migraine and its implications for public health and health policy. Nat Rev Neurol.

[6] Chaudhary R, Saini R, Rawat RS, Bachhas R, Majani R, Arya MH (2022). Migraine: evolution of a common disorder. Int J Sci Res Sci Technol.

[7] Steiner TJ, Stovner LJ, Birbeck GL (2013). Migraine: the seventh disabler. J Headache Pain.

[8] Akerman S, Goadsby PJ (2015). Neuronal PAC1 receptors mediate delayed activation and sensitization of trigeminocervical neurons: relevance to migraine. Sci Transl Med.

[9] Karatas H, Erdener SE, Gursoy-Ozdemir Y, Lule S, Eren-Koçak E, Sen ZD, Dalkara T (2013). Spreading depression triggers headache by activating neuronal Panx1 channels. Science.

[10] Stearns SA, Xun H, Haddad A, Rinkinen J, Bustos VP, Lee BT (2023). Therapeutic options for migraines in the microsurgical patient: a scoping review. [PREPRINT]. Plast Reconstr Surg.

[11] Kacperski J, Green A, Qaiser S (2020). Management of chronic migraine in children and adolescents: a brief discussion on preventive therapies. Paediatr Drugs.

[12] Nierenburg Hdel C, Ailani J, Malloy M, Siavoshi S, Hu NN, Yusuf N (2015). Systematic review of preventive and acute treatment of menstrual migraine. Headache.

[13] American Headache Society (2019). The American Headache Society position statement on integrating new migraine treatments into clinical practice. Headache.

[14] Thorlund K, Mills EJ, Wu P, Ramos E, Chatterjee A, Druyts E, Goadsby PJ (2014). Comparative efficacy of triptans for the abortive treatment of migraine: a multiple treatment comparison meta-analysis. Cephalalgia.

[15] Leroux E, Buchanan A, Lombard L (2020). Evaluation of patients with insufficient efficacy and/or tolerability to triptans for the acute treatment of migraine: a systematic literature review. Adv Ther.

[16] Amirlak B, Sanniec K, Pezeshk R, Chung M (2016). Anatomical regional targeted (ART) BOTOX injection technique: a novel paradigm for migraines and chronic headaches. Plast Reconstr Surg Glob Open.

[17] Brown BL, Craycraft LK, Justice SB (2020). Valproic acid in the treatment of migraines. Adv Emerg Nurs J.

[18] Rubio-Beltran E, Chan KY, Danser AJ, MaassenVanDenBrink A, Edvinsson L (2020). Characterisation of the calcitonin gene-related peptide receptor antagonists ubrogepant and atogepant in human isolated coronary, cerebral and middle meningeal arteries. Cephalalgia.

[19] de Vries T, Boucherie DM, van den Bogaerdt A, Danser AH, MaassenVanDenBrink A (2023). Blocking the CGRP receptor: differences across human vascular beds. Pharmaceuticals (Basel).

[20] Hutchinson S, Dodick DW, Treppendahl C, Bennett NL, Yu SY, Guo H, Trugman JM (2021). Ubrogepant for the acute treatment of migraine: pooled efficacy, safety, and tolerability from the ACHIEVE I and ACHIEVE II phase 3 randomized trials. Neurol Ther.

[21] Puledda F, Younis S, Huessler EM, Haghdoost F, Lisicki M, Goadsby PJ, Tassorelli C (2023). Efficacy, safety and indirect comparisons of lasmiditan, rimegepant, and ubrogepant for the acute treatment of migraine: a systematic review and network meta-analysis of the literature. Cephalalgia.

[22] Urits I, Jones MR, Gress K, Charipova K, Fiocchi J, Kaye AD, Viswanath O (2019). CGRP antagonists for the treatment of chronic migraines: a comprehensive review. Curr Pain Headache Rep.

[23] Hutchinson S, Silberstein SD, Blumenfeld AM, Lipton RB, Lu K, Yu SY, Severt L (2021). Safety and efficacy of ubrogepant in participants with major cardiovascular risk factors in two single-attack phase 3 randomized trials: ACHIEVE I and II. Cephalalgia.

[24] Puledda F, Tassorelli C, Diener HC (2023). New migraine drugs. Cephalalgia.

[25] Chiang CC, VanderPluym JH (2021). Ubrogepant in the acute management of migraine: a narrative review. J Pain Res.

[26] Dodick DW, Lipton RB, Ailani J, Lu K, Finnegan M, Trugman JM, Szegedi A (2019). Ubrogepant for the treatment of migraine. N Engl J Med.

[27] Bhakta M, Vuong T, Taura T, Wilson DS, Stratton JR, Mackenzie KD (2021). Migraine therapeutics differentially modulate the CGRP pathway. Cephalalgia.

[28] Zhang J, Gao LZ, Chen YJ (2019). Continuum of host-gut microbial co-metabolism: host CYP3A4/3A7 are responsible for tertiary oxidations of deoxycholate species. Drug Metab Dispos.

[29] Tfelt-Hansen P, Hüsing A, Diener HC (2021). Critique of the analysis of the time course for the antimigraine effect of ubrogepant 50 mg. Clinical relevance versus statistical significance. Cephalalgia.

[30] Goadsby PJ, Blumenfeld AM, Lipton RB (2021). Time course of efficacy of ubrogepant for the acute treatment of migraine: clinical implications. Cephalalgia.

[31] Boinpally R, Lu K (2022). Single-dose pharmacokinetics and safety of ubrogepant in adults with hepatic impairment: results from an open-label, phase 1 trial. Clin Pharmacol Drug Dev.

[32] Lipton RB, Dodick DW, Goadsby PJ (2022). Efficacy of ubrogepant in the acute treatment of migraine with mild pain vs moderate or severe pain. Neurology.

[33] Kish T (2018). Emerging therapies for patients with difficult-to-treat migraine. P T.

[34] Edvinsson L (2022). Calcitonin gene-related peptide (CGRP) is a key molecule released in acute migraine attacks-Successful translation of basic science to clinical practice. J Intern Med.

[35] Deen M, Correnti E, Kamm K (2017). Blocking CGRP in migraine patients - a review of pros and cons. J Headache Pain.

[36] Begasse de Dhaem O, Takizawa T, Dodick DW (2023). Long-term open-label and real-world studies of lasmiditan, ubrogepant, and rimegepant for the acute treatment of migraine attacks. Cephalalgia.

[37] Yadgarov IS, Filatova EG, Golubev VL, Berdnikova A V (2022). Relief of migraine attack — hepants. Russ Neurol J.

[38] Zhang H, Zhang XM, Zong DD, Ji XY, Jiang H, Zhang FZ, He SD (2021). miR-34a-5p up-regulates the IL-1β/COX2/PGE2 inflammation pathway and induces the release of CGRP via inhibition of SIRT1 in rat trigeminal ganglion neurons. FEBS Open Bio.

[39] Dabertrand F, Harraz OF, Koide M (2021). PIP2 corrects cerebral blood flow deficits in small vessel disease by rescuing capillary Kir2.1 activity. Proc Natl Acad Sci U S A.

[40] Lipton RB, Dodick DW, Ailani J, Lu K, Finnegan M, Szegedi A, Trugman JM (2019). Effect of ubrogepant vs placebo on pain and the most bothersome associated symptom in the acute treatment of migraine: the ACHIEVE II randomized clinical trial. JAMA.

[41] Voss T, Lipton RB, Dodick DW (2016). A phase IIb randomized, double-blind, placebo-controlled trial of ubrogepant for the acute treatment of migraine. Cephalalgia.

[42] Johnston KM, Powell L, Popoff E, Harris L, Croop R, Coric V, L'Italien G (2022). Rimegepant, ubrogepant, and lasmiditan in the acute treatment of migraine examining the benefit-risk profile using number needed to treat/harm. Clin J Pain.

[43] Yang CP, Liang CS, Chang CM (2021). Comparison of new pharmacologic agents with triptans for treatment of migraine: a systematic review and meta-analysis. JAMA Netw Open.

[44] Agostoni EC, Barbanti P, Calabresi P (2019). Current and emerging evidence-based treatment options in chronic migraine: a narrative review. J Headache Pain.

[45] Edvinsson L (2021). CGRP and migraine: from bench to bedside. Rev Neurol (Paris).

[46] Do TP, Guo S, Ashina M (2019). Therapeutic novelties in migraine: new drugs, new hope?. J Headache Pain.

[47] Leung L, Liao S, Wu C (2021). To probe the binding interactions between two FDA approved migraine drugs (ubrogepant and rimegepant) and calcitonin-gene related peptide receptor (CGRPR) using molecular dynamics simulations. ACS Chem Neurosci.

[48] Scott LJ (2020). Ubrogepant: first approval. Drugs.

[49] Johnston K, Popoff E, Deighton A (2022). Comparative efficacy and safety of rimegepant, ubrogepant, and lasmiditan for acute treatment of migraine: a network meta-analysis. Expert Rev Pharmacoecon Outcomes Res.

[50] Nedd M, Garland S, Falk N, Wilk A (2022). Ubrogepant: an oral calcitonin gene-related peptide (CGRP) receptor antagonist for abortive migraine treatment. Ann Pharmacother.

[51] Li CC, Dockendorf M, Kowalski K (2018). Population PK analyses of ubrogepant (MK-1602), a CGRP receptor antagonist: enriching in-clinic plasma PK sampling with outpatient dried blood spot sampling. J Clin Pharmacol.

[52] Zhang Z, Shu Y, Diao Y, Du Y, Chen L, Liu Y, Du B (2021). Calcitonin gene-related peptide receptor antagonist ubrogepant for the treatment of acute migraine: a meta-analysis. Medicine (Baltimore).

[53] Tepper SJ (2018). History and review of anti-calcitonin gene-related peptide (CGRP) therapies: from translational research to treatment. Headache.

[54] Negro A, Koverech A, Martelletti P (2018). Serotonin receptor agonists in the acute treatment of migraine: a review on their therapeutic potential. J Pain Res.

[55] Aronson JK (2016). Triptans. Meyler's Side Effects of Drugs: The International Encyclopedia of Adverse Drug Reactions and Interactions.

[56] Macone AE, Perloff MD (2017). Triptans and migraine: advances in use, administration, formulation, and development. Expert Opin Pharmacother.

[57] Law S, Derry S, Moore RA (2013). Triptans for acute cluster headache. Cochrane Database Syst Rev.

[58] Ailani J, Lipton RB, Hutchinson S (2020). Long-term safety evaluation of ubrogepant for the acute treatment of migraine: phase 3, randomized, 52-week extension trial. Headache.

[59] Marcus SC, Shewale AR, Silberstein SD, Lipton RB, Young WB, Viswanathan HN, Doshi JA (2020). Comparison of healthcare resource utilization and costs among patients with migraine with potentially adequate and insufficient triptan response. Cephalalgia.

[60] Chiang CC, Schwedt TJ (2020). Calcitonin gene-related peptide (CGRP)-targeted therapies as preventive and acute treatments for migraine—the monoclonal antibodies and gepants. Prog Brain Res.

[61] Moreno-Ajona D, Villar-Martínez MD, Goadsby PJ (2022). New generation gepants: migraine acute and preventive medications. J Clin Med.

[62] Dodick DW (2019). CGRP ligand and receptor monoclonal antibodies for migraine prevention: evidence review and clinical implications. Cephalalgia.

[63] Huang T, Xu Y, Chen Y, Bian J, Chu Z, Zhao S, Ma L (2022). Efficacy and safety of calcitonin gene-related peptide antagonists in migraine treatment: a meta-analysis. Brain Behav.

[64] Fda Fda, Cder Cder (2023). UBRELVY - ubrogepant tablet. https://dailymed.nlm.nih.gov/dailymed/drugInfo.cfm?setid=fd9f9458-fd96-4688-be3f-f77b3d1af6ab.

[65] Woodhead JL, Siler SQ, Howell BA, Watkins PB, Conway C (2022). Comparing the liver safety profiles of 4 next-generation CGRP receptor antagonists to the hepatotoxic CGRP inhibitor telcagepant using quantitative systems toxicology modeling. Toxicol Sci.

